# *In vitro Anti-Toxoplasma gondii* Activity of Root Extract/Fractions of *Eurycoma longifolia* Jack

**DOI:** 10.1186/1472-6882-12-91

**Published:** 2012-07-10

**Authors:** Nowroji Kavitha, Rahmah Noordin, Kit-Lam Chan, Sreenivasan Sasidharan

**Affiliations:** 1 Institute for Research in Molecular Medicine (INFORMM), Universiti Sains Malaysia, 11800 USM, Pulau Pinang, Malaysia; 2 School of Pharmaceutical Sciences, Universiti Sains Malaysia, USM 11800, Pulau Pinang, Malaysia

**Keywords:** *Toxoplasma gondii*, *Eurycoma longifolia*, Antiparasite, Toxoplasmosis, Toxoplamacidal activity

## Abstract

**Background:**

*Toxoplasma gondii* infection causes toxoplasmosis, an infectious disease with worldwide prevalence. The limited efficiency of drugs against this infection, their side effects and the potential appearance of resistant strains make the search of novel drugs an essential need. We examined *Eurycoma longifolia* root extract and fractions as potential sources of new compounds with high activity and low toxicity. The main goal of this study was to investigate the anti-*T. gondii* activity of crude extract (TACME) and four fractions (TAF 273, TAF 355, TAF 191 and TAF 401) from *E. longifolia*, with clindamycin as the positive control.

**Methods:**

*In vitro* toxoplasmacidal evaluation was performed using Vero cells as host for *T. gondii*. Light microscopy technique was used to study *in situ* antiparasitic activity.

**Results:**

Significant anti-*T. gondii* activity was observed with clindamycin (EC50 = 0.016 μg/ml), follow by TAF 355 (EC50 = 0.369 μg/ml) and TAF 401 (EC50 = 0.882 μg/ml). Light microscopy revealed that most Vero cells were infected after 3 h of exposure to *T. gondii*. After 36 h of exposure to the *E. longifolia* fraction, the host Vero cells showed no visible intracellular parasite and no remarkable morphological changes.

**Conclusions:**

Our study demonstrated that TAF 355 and TAF401 fractions may be the sources of new anti-*T. gondii* compounds.

## Background

Parasitic diseases still cause a major challenge to human well-being, particularly in poor populations living in tropical and subtropical climates with low-income economies [1]. Some conventional drugs are unaffordable for them and health facilities are also inaccessible. One of the common infections in tropical and subtropical climates is toxoplasmosis caused by *Toxoplasma gondii*. It is one of the most widespread protozoan parasites, chronically infecting approximately 30% of the global human population [2]. *T. gondii* causes severe neurological deficits in immunosuppressed patients (such as those with AIDS) and lymphadenopathy in healthy adults. It can cross the placenta (generally in women with no or low antibody levels) and cause congenital infections characterized by intra-cerebral calcifications, chorioretinitis, hydrocephaly or microcephaly, and convulsions [3].

Anti*-T. gondii* agents consisting of a combination of pyrimethamine and sulphonamides, especially sulphadiazine, are still most frequently used, and they inhibit dihydrofolate reductase, a key enzyme in the synthesis of purines [4,5]. Other alternative drugs include clindamycin, atovaquone and spiramycin. The treatment of *T. gondii* infections, in general, accentuates the problem of the limited effectiveness of the available anti-parasitic agents, their side effects and the potential appearance of resistant strains. Other options for the toxoplasmosis treatment are desirable, thus the search for new anti-parasitic agents is needed.

The need for fundamental research [6] on anti-*T. gondii* agents leads our study. On the other hand, the choice of using *E. longifolia* as source was based on previous reports activity against *Plasmodium* parasite. Like *T. gondii*, *Plasmodium* is an apicomplexan intracellular protozoa, therefore *E. longifolia* can also be a potential source of anti-*T. gondii* agents.

*E. longifolia* Jack, from the Simaroubaceae family and identified locally as ‘Tongkat Ali’ or ‘Pasakbumi’ has been commonly prescribed in traditional medicine as a febrifuge and a remedy for dysentery, glandular swelling and fever [7,8]. *E. longifolia* is found in primary and secondary, evergreen and mixed deciduous forests in Burma, Indochina, Thailand, Malaysia, Sumatra, Borneo and Philippines. It is popularly sought after as a singly or an essential component for the treatment of fevers, aches, sexual insufficiency and also as health supplements. Traditional medicinal users usually take a decoction of the roots in water as a health tonic and anti-stress remedy. Extracts derived from the roots of this plant were also found to demonstrate activity when evaluated with the sarcoma 180 model [9]. Moreover, anti-malarial [10-14] and cytotoxic [15-18] activities were also reported being linked to the presence of quassinoids, squalene derivatives, biphenyl-neolignans, tirucallane-type triterpenes, canthine-6-one and carboline alkaloids. Specially, three quassinoids, eurycomanone, its 13α(21)-epoxy and 13, 21-dihydro analogues were identify as having greater anti-plasmodial activity [19].

## Methods

### Plant material

The roots of *E. longifolia* Jack were identified and purchased in Perak, Malaysia by a pharmaceutical company, Hovid Berhad, in Ipoh. A voucher specimen (No. 785–117) was deposited in Penang Botanical Garden, Penang, Malaysia.

### Extraction and isolation

The air-dried powdered roots of *E. longifolia* was extracted with 6 × 4 L of 95% methanol for 6 days at 60°C. The combined methanol extract was then evaporated to dryness to yield a dark brown residue. Subsequently, this dark brown residue was chromatographed on a resin column with several alcohol/water mixtures to yield four fractions (Fr 1–4) such as alcohols layers, water layer and residue layers. The four fractions were concentrated under vacuum and were resuspended in water and then partitioned successively with saturated *n*-butanol to yield several sub-fractions. Successive column chromatography using silica gel and centrifugal thin-layer chromatography of the sub-fractions with various CHCl_3_-methanol mixtures yielded the desired four active sub-fractions fractions (TAF 273, TAF 355, TAF 191 and TAF 401). The fractions that contained TAF 273, TAF 355, TAF 191 and TAF 401 were identified by TLC comparison. One methanol extracts (TACME) and four fractions (TAF 273, TAF 355, TAF 191 and TAF 401) were used in this study. The RPMI-1640 medium was used as the solvent for preparation of different dilutions of the plant extracts.

### *Toxoplasma gondii* strain

The experimental procedures relating to the animals were authorized by Universiti Sains Malaysia Ethical committee (USM/ PPSF50(003)JLD2) before starting the study and were conducted under the internationally accepted principles for laboratory animal use and care. Tachyzoites from the virulent RH strain of *T. gondii* were maintained by intraperitoneal passages in Swiss albino mice and collected in phosphate-buffered saline (PBS), pH 7.2, at 3–4 day intervals. The ascites fluid obtained from infected mice was centrifuged at 200 × *g* for 10 min at room temperature to remove host cells and debris. The supernatant, which contained the parasites, was collected and centrifuged at 1000 × *g* for 10 min. The pellet was washed with PBS, pH 7.2 and then in RPMI medium without foetal bovine serum (FBS). The parasites were used within 30–40 min of their removal from the mice peritoneal cavity and the viability was evaluated using the trypan blue dye-exclusion test.

### Host cells

The results of our previous study indicated that *E. longifolia* fractions did not have a significant effect on Vero cell growth, and *E. longifolia* fractions can be used safely for the anti-*Toxoplasma* assay [20]. The cell line was initiated from kidney of a normal adult African green monkey on March 27th, 1962, by Yasummura and Kawakita at the Chiba University, Japan (American Public Health Association, 1992). Vero cells were maintained in RPMI-1640 medium supplemented with 10% FBS, glutamine (2 raM), penicillin (100 units/ml) and streptomycin (100 μg/ml). The cells were cultured at 37°C in a humidified 5% CO_2_ incubator.

### Assay of toxoplasmacidal activity

*In vitro* toxoplasmicidal studies were carried out using the method described by Cover and Gutteridge [21]. Briefly, 45 μl of tachyzoites suspension containing 10^6^ cells/ml were incubated, with 5 mL of each fractions/clindamycin (dissolved in DMSO [1%w/v] at final concentration of 1.56–100 μg/ml) [22] at 37°C in 96 wells micro plates. Tachyzoites vitality was determined under an inverted microscope with the trypan blue dye exclusion test after 24 h of incubation in 10% CO_2_ chamber. The results were expressed as % mortality. Clindamycin (Sigma, USA), a drug that has been used in human and animal anti-*T. gondii* therapy was used as the positive control [23]. The extract/fractions were assayed in triplicate at each concentration.

### *In vitro* infection and effectors

Vero cells were harvested during exponential growth (day 2) and cultured in 96-well plates (ca. 6 × 10^4^ cells/ml). Then, 3 × 10^5^ parasites/ml were added to each well (parasite: cell ratio = 5:1, final volume 200 μl) [24]. Six hours after inoculation, the infected cells were washed twice with RPMI 1640 medium without FBS to remove any non-adherent parasites. After 18 h incubation, RPMI 1640 medium supplemented with 2% FBS was added to each well along with different concentrations of the fractions/ clindamycin (at final concentration of 1.56–100 μg/ml) [22]. After 24 h of treatment, anti-*T. gondii* activity and cytotoxicity of the extracts were examined using an MTS (3-(4,5-dimethylthiazol-2-yl)-5-(3-carboxymethoxyphenyl)-2-(4-sulfophenyl)-2 H-tetrazolium, inner salt) (Promega, Madison, WI) assay [25]. The assay was conducted in 96-well plates and assayed using a microplate reader (Spectra Max Gemini XG, parameters chosen in SOFT max pro 4.0; Molecular Devices, Sunnyvale, CA) using a wavelength of 490 nm. Fraction TAF 355 and TAF 401 which showed the best toxoplasmacidal activity were assayed in triplicate at each concentration in this study. All data points represent the mean of three independent experiments. The median effective concentration (EC_50_) value refers to the concentration of the fractions/ clindamycin necessary to inhibit 50% of the control values. Selectivity refers to the mean of the EC_50_ value for Vero cells relative to the mean of the EC_50_ value of the *T. gondii*[26].

### Light microscope observation of the tachyzoites in cell lines

Cell line was cultured on a glass cover slip in a 35 mm cell culture dish until confluent, and then infected with 1 × 10^4^ tachyzoites/dish. After incubation for 4 h, the monolayers were washed with Hanks balanced salt solution (HBSS; Gibco Inc., USA) and the fractions in RPMI medium were added. The glass cover slips were taken from the dishes at 0, 1, 2, 3, 4, 5, 6, 8, 10, 12, 24, or 36 h after adding the TAF 355, TAF 401, clindamycin and 1% DMSO (negative control). All the glass cover slips were washed with HBSS and fixed by methanol prior to staining with Giemsa (Sigma Inc., USA). All the prepared samples were observed under oil lens at 1000× magnification on a light microscope, and the images were captured by using the camera and software [22].

### Statistical analysis

All values are expressed as the mean ± S.D. of three measurements. Statistical analysis was performed using ANOVA followed by Tukey's Honestly Significant Differences (HSD) using SPSS software. Significance was assumed as *p* < 0.05. Probit analysis of mortality data from MTS assay was conducted using SPSS (ver10.0) computer software (SPSS for Windows, SPSS Inc., 1997). The Probit analysis of mortality output was used to calculate the LC_50_ value.

## Results

### Toxoplasmacidal activity

**Figure 1:**
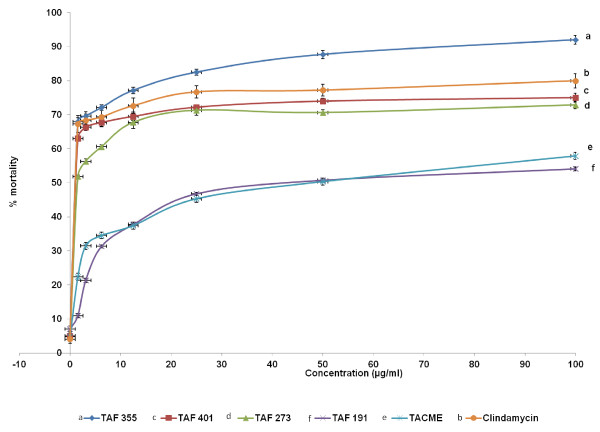
**Effects of*E. longifolia*fractions on the proliferation of*T. gondii*tachyzoites.**

The results of *in vitro* anti-*T. gondii* activity against *T. gondii* RH strain and selectivity are summarised in Table 1 and Figure 1. Toxoplasmacidal activity was found in all the samples tested and mortality was observed in the following order (P < 0.05): TAF 355 > clindamycin > TAF 401 > TAF 273 > TACME > TAF 191 (Figure 1). Clindamycin had high anti-*T. gondii* activity (EC50 = 0.016 μg/ml and selectivity = 656.3) relative to TAF 355 (EC50 = 0.369 μg/ml and selectivity = 63.7) and TAF 401 (EC50 = 0.882 μg/ml and selectivity = 17.0). Based on these results TAF 355 and TAF 401 were used for further *in-vitro* anti-*Toxoplasma* activity evaluation.

**Table 1:** ***In vitro*anti-*T. gondii*RH strain activity and selectivity of*E. longifolia*fractions and clindamycin**

**Tested drugs name**	**EC**_**50**_**(μg/ml)**	**Selectivity**^**a**^
Fractions TAF 355	0.369	63.7
Fractions TAF 401	0.882	17.0
Clindamycin (Positive control)	0.016	656.3

EC_50_, median effective concentration.

^a^ Ratio of the EC_50_ value for Vero cells to the EC50 value for *Toxoplasma gondii* tachyzoites. The EC50 value for Vero cells was used from our previously published data (TAF 35 = 23.50 μg/ml, TAF 401 = 15.00 μg/ml and Clindamycin = 10.5 μg/ml) [20].

### *In vitro* infection and effectors

**Figure 2:**
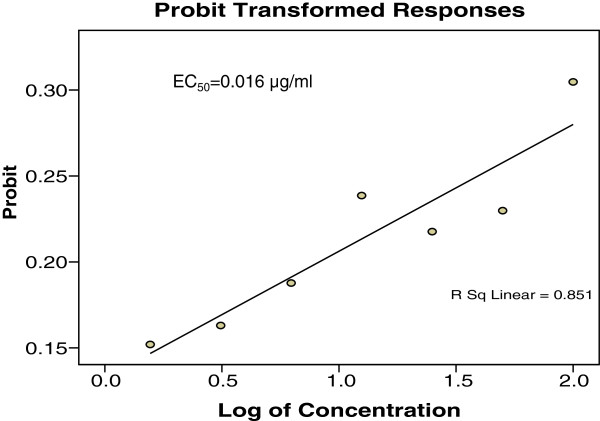
***In vitro*infection and effectors of Clindamycin against*T. gondii*tachyzoites in infected Vero cell lines after 24 hours incubation.**

**Figure 3:**
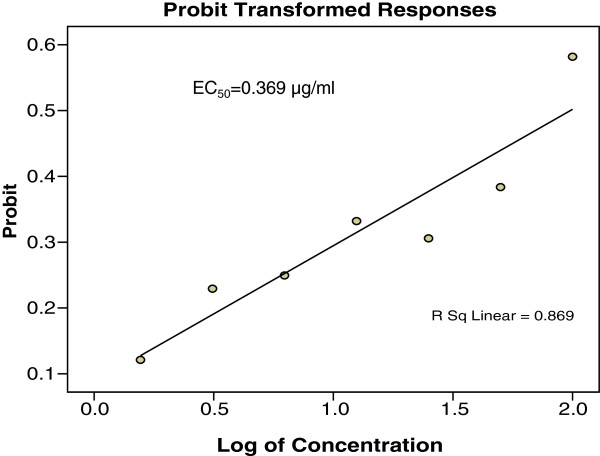
***In vitro*infection and effectors of*E. longifolia*TAF 355 fraction against*T. gondii*tachyzoites in infected Vero cell lines after 24 hours incubation.**

**Figure 4:**
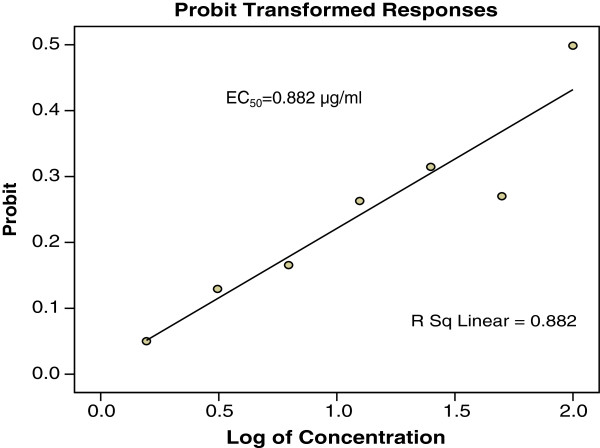
***In vitro*infection and effectors of*E. longifolia*TAF 401 fraction against*T. gondii*tachyzoites in infected Vero cell lines after 24 hours incubation.**

Figures 2, 3 and 4 showed the effects of TAF 355, TAF 401 and clindamycin on infected cells with *T. gondii*. At each concentration TAF 355 and TAF 401 inhibited *T. gondii* tachyzoites in Vero cells in a concentration-dependent manner. Even at low concentrations TAF 355 and TAF 401 dramatically inhibited *T. gondii* tachyzoites in Vero cells. TAF 355 showed the greatest inhibition on *T. gondii* tachyzoites growth in Vero cells with lowest EC_50_ value (0.369 μg/ml) followed by TAF 401 (0.882 μg/ml).

### Observing the effect of anti-*Toxoplasma gondii* under microscope

**Figure 5:**
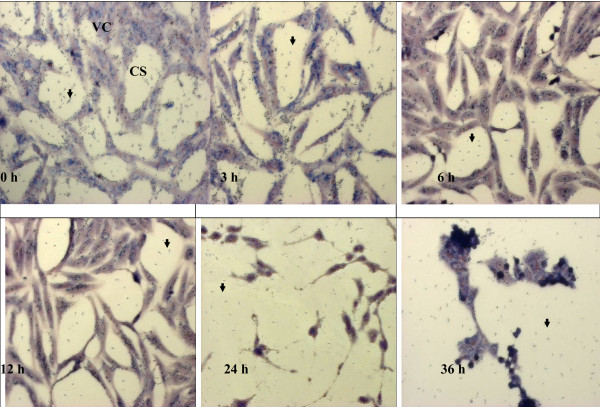
**Morphology of Vero cells infected with*T. gondii*with 1% DMSO (negative control) treatment stained using Giemsa staining.** The tachyzoites (↓) can be seen inside the Vero cells (VC), as well as adhered to the glass cover slip (CS).

**Figure 6:**
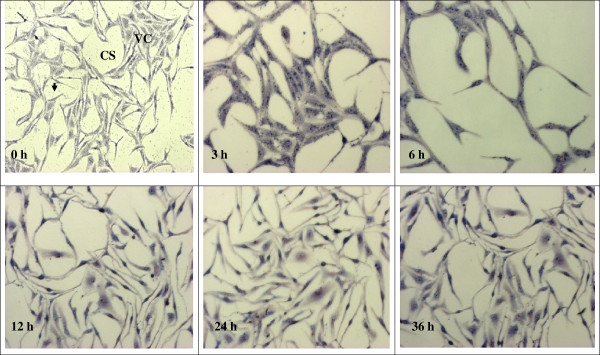
**Morphology of Vero cells infected with*T. gondii*and clindamycin-treatment stained using Giemsa staining.** The tachyzoites (↓) can be seen inside the Vero cells (VC), as well as adhered to the glass cover slip (CS).

**Figure 7:**
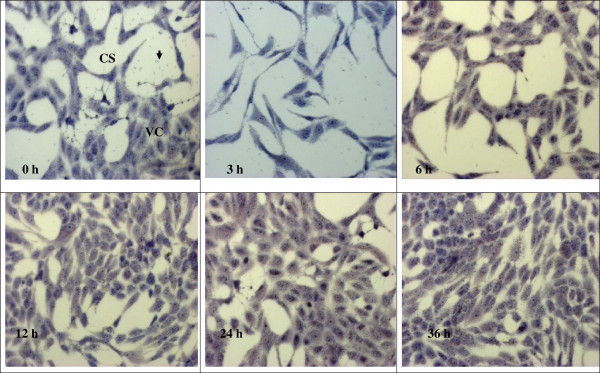
**Morphology of Vero cells infected with*T. gondii*and TAF 355-treatment stained using Giemsa staining.** The tachyzoites (↓) can be seen inside the Vero cells (VC), as well as adhered to the glass coverslip (CS).

**Figure 8:**
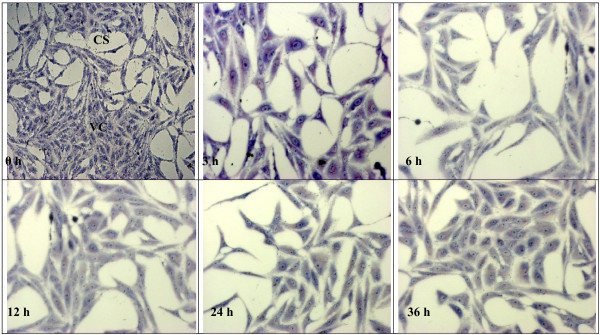
**Morphology of Vero cells infected with*T. gondii*and TAF 401-treatment stained using Giemsa staining.** The tachyzoites (↓) can be seen inside the Vero cells (VC), as well as adhered to the glass coverslip (CS).

The observation of the effects of 1% DMSO, clindamycin, TAF 355 and TAF 401 under light microscope on *T. gondii* in Vero cells are shown in the Figures 5, 6, 7 and 8. After infection for 3 h, many parasites were observed inside the Vero cells (Figure 5). Tachyzoites can be seen inside the Vero cells as well as adhered to the glass coverslip in the negative control group treated with 1% DMSO, even at 36 h. The morphology of Vero cells were changed remarkably in the negative control group with lowest confluence of Vero cells at 36 h. However, when 100 μg/ml clindamycin, TAF 355 and TAF 401 were added (Figure 6, 7 and 8) the number of tachyzoites decreased sharply. Merely 3 h after adding the drugs or fractions, there were few visible parasites in the infected cells. After 6 h, *T. gondii* tachyzoites were rarely seen in the infected cells, which continued to grow. Vero cells did not change remarkably after exposure to TAF 355 and TAF 401 for 36 h compared to the positive control. In addition, the degree of confluence of Vero cells exposed to TAF 355 was highest, followed by TAF 401, and lowest confluence was seen for the cells treated with clindamycin, as expected.

## Discussion

Diseases caused by tropical parasites affect hundreds of millions of people worldwide but have been largely neglected for drug development because they only affect people in poor regions of the world [27]. Hence the development of cheap, reliable and affordable drugs to address this problem is of paramount importance. The discovery of anti-parasitic drugs requires investigation of molecules with the ability to kill parasites but not their hosts. Recently, greater emphasis has been given towards the studies on complementary and alternative medicine that deals with parasitic disease management. Several studies have been conducted on herbs under a multitude of ethno botanical grounds. While the pharmaceutical industry has a long track record of success in developing natural product drugs for the parasitic diseases market, for more than half a century there has also been an active interest in the systematic screening of extracts from medicinal plants and other organisms for their potential anti-parasitic properties. Therefore, the current study was conducted to develop a new anti-*Toxoplasma* agent from our local medicinal plant *E. longifolia*.

The anti-*T. gondii* activity of *E. longifolia* has not been reported before. In this study, we found that *E. longifolia* fractions significantly inhibited *T. gondii* growth, even at low concentrations (0.369 μg/ml). The most potent anti–*T. gondii* activity was found in TAF 355, which showed an EC50-value of 0.369 μg/ml, indicating a good anti–*T. gondii* activity. In addition, TAF 355 showed high anti-*T. gondii* activity but had no toxicity against the host cells. This finding was proved in our previous study where one extract (TACME) and four fractions (TAF 273, TAF 355, TAF 191 and TAF 401) from *E. longifolia* root were evaluated for their *in vitro* cytotoxicity activity against Vero cells. The results of this study suggest that TAF 401 showed lower activity and the TAF 355 fraction did not cause any toxicity against Vero cell lines tested. The *in vitro* infected models used for anti-*T. gondii* activity in this study are fundamental to anti-*Toxoplasma* drug research [28,29] and worked well in this study. Vero cells were used for culturing *T. gondii in vitro* in this study. The addition of *E. longifolia* fractions to monolayers of Vero cells showed that they remained metabolically active and viable after infected with *T. gondii*. In our study, we confirmed that *T. gondii* could invade Vero cells and proliferate quickly. Our study demonstrated that TAF 355 effectively inhibited the growth of *T. gondii*, and was less toxic to Vero cells than Clindamycin. This finding signifies that TAF 355 might be potential candidate as an alternative to Clindamycin for the treatment of toxoplasmosis which deserves further study. Additionally to its toxicity, Clindamycin is expensive, limiting its availability to poor populations particularly living in the tropical and subtropical climates with low-income economies.

Similar results were also reported by Choi et al. [30]. They used 15 traditional herbs methanol extracts to evaluate the anti-*T. gondii* activity by using HeLa cells as host. The results show that the herbal extracts exhibited the best activity with the EC50 values ranged from 0.11 mg/mL to 2.28 mg/mL. They also reported that *Zingiber officinale* extracts (EC(50) = 0.18 mg/mL), displayed the highest selective toxicity (selectivity = 10.1) and *Sophora flavescens* Aiton extracts showed the highest anti-*T. gondii* activity (EC(50) = 0.20 mg/mL) with high selective toxicity.

Advances in microscopy technique to observed at ultrastructural level of cells morphology enhance the understanding of *in situ* antiparasitic activity observation. The microscopy method enables *in situ* observations of the effect of anti-parasitic agents on the organisms [31]. In this study light microscopy technique was used to observe the suppression of *T. gondii* growth by clindamycin, TAF 355 and TAF 401. The tachyzoites can be seen inside the Vero cells, as well as adhered to the glass coverslip. As predicted, the number of tachyzoites observed in the presence of clindamycin, TAF 355 and TAF 401 decreased in a time-dependent manner with increasing time of incubation in the medium with clindamycin. The morphology of Vero cells changed remarkably after the exposure to clindamycin, this exemplified the previous observations that commercial drugs may have severe side effects on the host cells [32]. Normally the rapidly proliferating *T. gondii* tachyzoites propagate by host cell lysis, egression, reattachment, and invasion of new host cells [33]. The remarkable changes observed in the morphology and confluence of Vero cells in 1% DMSO treated group (negative control) is probably due to the above fact. However, TAF 355 and TAF 401 treatment did not remarkably affect the normal growth and morphology of the infected host cells despite the potent anti–*T. gondii* activities, the cells seemed to remain metabolically active and viable. In fact cells proliferation and confluence were higher than when exposed to clindamycin. This observation verified that *E. longifolia* fractions had high anti-*T. gondii* activity but had no selective toxicity against the infected host cells, particularly TAF 355 and TAF 401. Our results also indicate that the mechanism of action of TAF 355 and TAF 401 against *T. gondii* differs from its activity against the host cells. Based on the above results, TAF 355 may be better than clindamycin for the treatment of toxoplasmosis.

With regard to the mechanism of action for TAF 355 and TAF 401 against *T. gondii*, we hypothesize that TAF 355 and TAF 401 may produce intracellular oxidative stress by an indirect mechanism [34]. Existing drugs such as atovaquone inhibit apicomplexans such as *Plasmodium falciparum* through redox mechanisms [35]. In contrast, the TAF 355 and TAF 401 protected the Vero cells. The exact mechanism of action of TAF 355 and TAF 401 at the level of the host cell remains unknown. However, the favorable effect may be attributed to its anti-oxidant properties, which have been well documented in the previous studies [36,37]. Mitochondria are the largest source of reactive oxygen species (ROS) within cells [38]. Moreover, uncontrolled superoxide flashes in mitochondria contribute to global oxidative stress, playing a key role in hypoxia/reoxygenation injury in cells [39]. This model provides a rational explanation for why TAF 355 and TAF 401 inhibit *T. gondii* growth and protects the Vero cells by selective toxicity. To confirm this, we will investigate the mechanism of action of TAF 355 and TAF 401 in our future studies.

## Conclusions

The search for anti-*T. gondii* agent from Malaysia medicinal plants has led to the finding that fractions of *E. longifolia*, in particular TAF 355, showed potent anti-*T. gondii* activity. These results, first reported in this work, have allowed us to propose that fractions from *E. longifolia* root are likely the sources of new compounds that could be used to treat *T. gondii* infections. Further studies will be necessary to identify, isolate and characterized these active compounds.

## Competing interest

The authors declare that they have no competing interests.

## Authors’ contributions

NK, RN, KLC and SS developed and piloted the survey. NK, RN, and SS performed the analysis. NK, RN, KLC and SS wrote the manuscript. All authors have read and approved the final version of the manuscript.

## Pre-publication history

The pre-publication history for this paper can be accessed here:

http://www.biomedcentral.com/1472-6882/12/91/prepub
